# Evaluation of insulin expression and secretion in genetically engineered gut K and L-cells

**DOI:** 10.1186/1472-6750-12-64

**Published:** 2012-09-19

**Authors:** Zalinah Ahmad, Mina Rasouli, Ahmad Zaid Fattah Azman, Abdul Rahman Omar

**Affiliations:** 1 Laboratory of Vaccines and Immunotherapeutics, Institute of Bioscience, Universiti Putra Malaysia, UPM Serdang, Selangor, 43400, Malaysia; 2 Department of Pathology, Faculty of Medicine and Health Sciences, Universiti Putra Malaysia, UPM Serdang, Selangor, 43400, Malaysia; 3 Department of Community Health, Faculty of Medicine & Health Sciences, Universiti Putra Malaysia UPM Serdang, Selangor, 43400, Malaysia

**Keywords:** Diabetes gene therapy, Insulin expression, K-cells, L-cells

## Abstract

**Background:**

Gene therapy could provide an effective treatment of diabetes. Previous studies have investigated the potential for several cell and tissue types to produce mature and active insulin. Gut K and L-cells could be potential candidate hosts for gene therapy because of their special features.

**Results:**

In this study, we isolated gut K and L-cells to compare the potential of both cell types to produce insulin when exposed to similar conditions. The isolated pure K and L-cells were transfected with recombinant plasmids encoding insulin and with specific promoters for K or L-cells. Insulin expression was studied in response to glucose or meat hydrolysate. We found that glucose and meat hydrolysate efficiently induced insulin secretion from K and L-cells. However, the effects of meat hydrolysate on insulin secretion were more potent in both cells compared with glucose. Results of enzyme-linked immunosorbent assays showed that L-cells secreted more insulin compared with K-cells regardless of the stimulator, although this difference was not statistically significant.

**Conclusion:**

The responses of K and L-cells to stimulation with glucose or meat hydrolysate were generally comparable. Therefore, both K and L-cells show similar potential to be used as surrogate cells for insulin gene expression *in vitro*. The potential use of these cells for diabetic gene therapy warrants further investigation.

## Background

Diabetes mellitus is a metabolic disorder associated with abnormally high blood glucose levels in patients. Untreated or improperly treated diabetes can result in acute complications that can lead to death or serious long-term complications. Therefore, several approaches have been considered to develop effective and convenient treatments for diabetes, such as gene therapy [1-3]. In the last decade, several gene therapy strategies have been developed to regulate physiologic blood glucose levels in patients with diabetes. One option that has been studied is to replace pancreatic β-cells with engineered non-pancreatic β-cells to generate potential target cells for gene therapy. These surrogate β-cells were able to synthesize and secrete active insulin; however, insulin secretion was not regulated by glucose, unlike endogenous β-cells [4-7]. Recent studies using enteroendocrine cells have shown the good potential for using these cells for diabetes gene therapy. This is primarily because of their ability to respond dynamically to a nutrient stimulus.

Enteroendocrine cells are located in the intestinal mucosa and secrete incretin hormones such as glucose-dependent insulinotropic peptide (GIP) and glucagon-like peptide-1 (GLP-1). GIP and GLP-1 are naturally secreted from gut K and L-cells, respectively. K-cells are mainly scattered in the duodenum whereas L-cells are predominantly found in the distal ileum. Both GIP and GLP-1 are rapidly secreted following food intake and return to the basal level following their degradation into inactive forms [8,9]. Many signaling factors including nutrients, neural elements, and hormones are capable of regulating the secretion of incretins although nutrients in the lumen strongly stimulate GIP and GLP-1 secretion [10,11]. It was reported that GIP and GLP-1 hormones induce insulin secretion from pancreatic β-cells to control blood glucose homeostasis. These two hormones are also involved in many other metabolic pathways. For example, GIP promotes β-cell proliferation, whereas GLP-1 potently inhibited glucagon secretion and induces gastric emptying and satiety [12].

Some properties of enteroendocrine cells make them appealing candidates for surrogate β-cells [13]. First, K and L-cells express prohormone convertases 2 and 3, which are required to process proinsulin to mature insulin [14]. Second, the expression of glucokinase and glucose transporter II in K and L-cells provides a glucose sensitive system similar to that of pancreatic β-cells [15]. Third, K and L-cells have cell-specific promoters like GIP and GLP-1, respectively. Finally, K and L-cells are easily accessible. Some research groups have studied insulin expression under the control of a GIP promoter and reported that K-cells secrete insulin in response to different nutrient stimuli at different levels *in vitro* and *in vivo*[16-18]. In other investigations, engineered L-cells were used as the host cells for insulin expression and viral promoters were used to express the insulin gene [19-21]. By contrast, the proglucagon promoter was used in our previous study, in which we examined the expression of insulin in L-cells [22]. These observations using K and L-cells raised a critical question concerning which intestinal cells should be used in the context of diabetes gene therapy for effective regulation of glucose homeostasis.

Therefore, the aim of this study was to evaluate and compare insulin expression efficiency in engineered K and L-cells maintained in identical conditions. To achieve this aim, pure K and L-cells were isolated from a heterogeneous population of intestinal cells (STC-1 cells) as basic models for gene expression studies. Then, we examined insulin expression in response to stimulation by glucose and meat hydrolysate (MH) in both cell types. The results of the expression studies provide useful information on the competency of both intestinal cell lines for synthesizing and secreting insulin. We found that meat hydrolysate is a potent stimulator of insulin expression in both types. Interestingly, we found no significant difference in insulin expression between the two cell types. Taken together, our results provide evidence to show that gut K and L-cells can be used to study gene expression or establish cell-based therapies. Our assessment of insulin gene expression in these cells also indicates that K and L-cells are potential candidate hosts for gene therapeutic treatment of diabetes or related disorders.

## Methods

### Plasmid construction

GIP/Ins/pBud and GLP-1/Ins/pBud plasmids were designed to establish insulin gene expression in K and L-cells, respectively. The GIP promoter and the insulin gene were inserted into the pBudCE4.1 vector (Invitrogen, Carlsbad, CA, USA) for the construction of the GIP/Ins/pBud plasmid. The GLP-1 promoter and insulin gene were used to construct the GLP-1/Ins/pBud plasmid. The nucleotides of the GIP promoter region were amplified from a rat-BAC clone (CH230-313 G4) obtained from the BACPAC Resource Center (Children’s Hospital Oakland Research Institute, Oakland, CA, USA). An upstream sequence of ~1,100 bp of the rat GIP gene is essential for promoter activity [23]. The nucleotides of proglucagon promoter region (approximately 2,300 bp) were amplified from the Glu.BS plasmid, which was kindly provided by Dr. Yvan Gosmain (University of Geneva, Geneva, Switzerland) [24]. The human insulin gene (approximately 1,800 bp) was amplified from human genomic DNA extracted from a blood donor.

We also constructed GIP/Neo/pBlu and GLP-1/Neo/pBlu plasmids, which were designed to isolate K and L-cells, respectively, from a heterogeneous population of intestinal cells. The GIP promoter and neomycin resistant gene were cloned into the pBluescript-II-SK vector (Stratagene, La Jolla, CA, USA) to construct the GIP/Neo/pBlu plasmid. The GLP-1 promoter and neomycin resistant gene were used to construct GLP-1/Neo/pBlu plasmid. The neomycin resistant gene (1,200 bp) was amplified from pcDNA3 plasmid (Invitrogen). A high fidelity PCR kit was used to amplify the PCR products. All of the enzymes and kits used in this study were purchased from Fermentas (Vilnius, Lithuania).

### Transfection and selection of stable K and L-cells

K and L-cells were isolated from STC-1 cell line, which were kindly supplied by Prof. Douglas Hanahan (University of California San Francisco, San Francisco, CA, USA). The STC-1 cells were grown in Dulbecco’s modified Eagle’s medium (DMEM) supplemented with 10% fetal bovine serum (fetal bovine serum) (PAA, Pasching, Austria) at 37°C, as previously described [22]. Cells were transfected using Lipofectamine-2000 (Invitrogen). To isolate pure K and L-cells, separate batches of STC-1 cells were transfected with the GIP/Neo/pBlu or GLP-1/Neo/pBlu plasmids. The transfected cells were treated with an antibiotic (400 μg/ml geneticin) for 2 weeks (Sigma, St Louis, MO, USA). Five stable K-cell clones and five stable L-cell clones treated with antibiotics were selected for propagation as pure cell lines.

To study gene expression ability and efficiency in these purified cells, we established insulin-producing K and L-cells by transfecting the cells with the GIP/Ins/pBud or GLP-1/Ins/pBud plasmids, respectively. The cells were incubated in medium containing an antibiotic (500 μg/ml zeocin) for 2 weeks. Five clones from each of the transfected cell types that survived the zeocin condition were selected for propagation and gene expression analyse. The optimal concentration of antibiotics was determined by an MTT assay, as previously described [22].

### Assessment of insulin secretion

We assessed the capacity for insulin secretion essentially as previously described [22]. All of the experimental conditions and cell culture factors were identical for both K and L-cells. Briefly, 3 × 10^6^ cells were seeded in six-well tissue culture plates. The cells were then incubated in basal media containing DMEM, 5 mM glucose, and 1% FBS overnight. The secretion test was started by replacing the medium with fresh basal medium for 2 h. Then, the cells were treated with (1) basal medium (containing 5 mM glucose), (2) basal medium supplemented with 25 mM glucose (containing 25 mM glucose), or (3) basal medium supplemented with 2% (w/v) meat hydrolysate. The cells and media were collected 1 h later to assess insulin gene expression.

### Quantitative polymerase chain reaction (Q-PCR)

Q-PCR was performed to confirm that the K and L-cells were efficiently isolated and to determine the insulin mRNA expression level. Total RNA was extracted from collected cells using an RNeasy plus mini kit (Qiagen, Hilden, Germany). The cDNA was synthesized by the iScript cDNA Synthesis Kit (Bio-Rad, Hercules, CA, USA) and Q-PCR was performed using specific primers. CFX Manager Software (version 1.0.1035.131; Bio-Rad) and a Bio-Rad thermal cycler were used to analyze the data. Gene expression was normalized for the expression of mouse β-actin and β-2 microglobulin.

### Enzyme-linked immunosorbent assay (ELISA)

A human insulin ELISA kit (ALPCO Diagnostics, Salem, NH, USA) was used to measure the amount of insulin that was secreted into the culture medium in each batch of cells. This ELISA kit has 100% cross reactivity with mature human insulin, whereas no reaction was reported with other forms, such as pro-insulin, C-peptide. Samples of cell culture medium for each treatment condition were measured.

### Statistical analysis

Statistical analyses were done using SPSS version 19 (SPSS Inc., Chicago, IL, USA). Insulin levels and the percentage increase in insulin secretion were treated as continuous variables. The percentage increase in insulin secretion was calculated as (post-induction insulin level – basal insulin level) / basal insulin level × 100. Normality of distribution was assumed based on observation of histograms and statistical tests. Equality of variance was also checked. Paired samples t-tests were used to compare the mean insulin levels between basal and post induction. Independent samples t-tests were used to compare the percentage increase in insulin secretion between each cell type and treatment. Confidence intervals and α were set at 95% and 0.05 respectively. For multivariate two-way analysis of variance, both cell type and stimulant type were included in the main effect models. There were no statistically significant interaction terms. All model assumptions, including normality, equal variance of residuals, model fitness, linear relationships, and absence of statistical interaction were tested and met.

## Results and discussion

### Isolation of stable intestinal K and L-cells

The STC-1 cell line is a heterogeneous population of intestinal cells that was first isolated and characterized by Rindi et al. 1990. They reported that this cell line consists of 7% K-cells, which express GIP, and 5% L-cells, which express GLP-1 [25]. Therefore, STC-1 cells have been used to isolate pure K and L-cells. In 2002, Ramshur et al. used a selective method to isolate K-cells from STC-1 cells [17]. Their method was designed to isolate pure cells from the heterogeneous STC-1 cells by establishing resistance to an antibiotic. To achieve this, an antibiotic resistance gene is expressed under the control of a specific promoter within the desired cells. The cells are then grown in a medium containing the antibiotic. The cells that recognize the promoter express the protein that confers antibiotic resistance and only these cells are able to survive the antibiotic treatment condition. We used the same method to isolate K and L-cells in this study. The recombinant GIP/Neo/pBlu plasmid, which includes a GIP promoter upstream of the neomycin resistant gene, was constructed to isolate pure K-cells from STC-1 cells following treatment with geneticin. The GIP promoter is specifically recognized by K-cells. Therefore, the stable cells that survived the antibiotic condition were isolated as pure K-cells. The same method was used to obtain pure L-cells [22].

**Figure 1:**
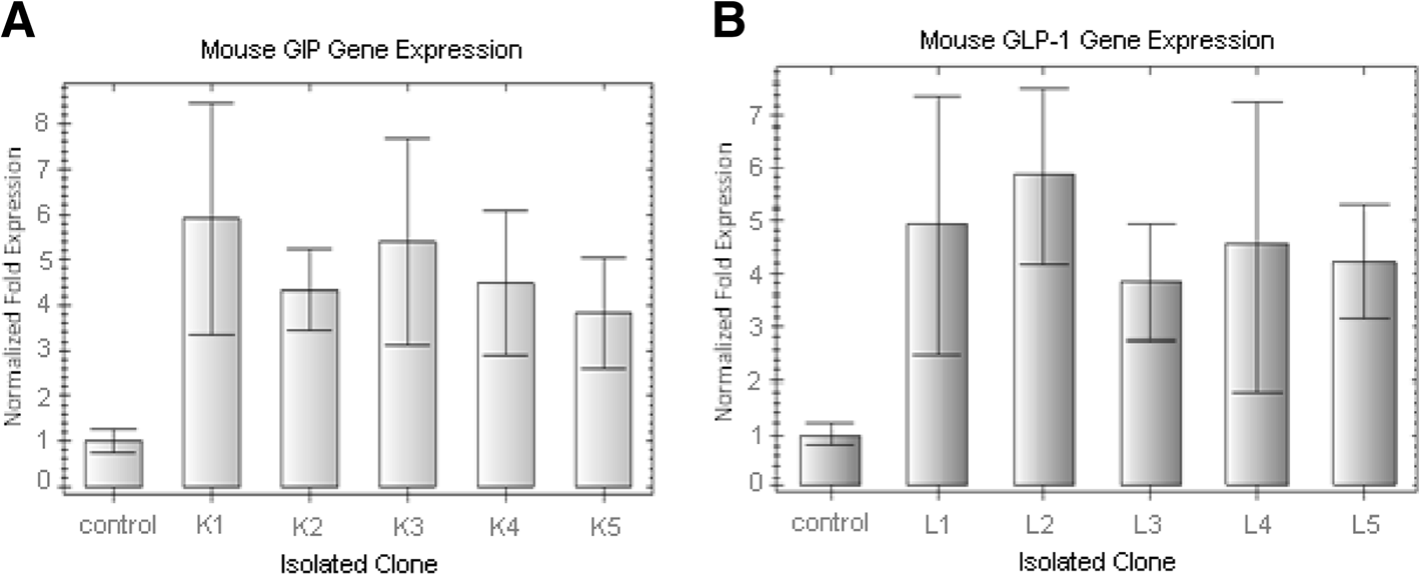
**Q-PCR analysis of the isolated clones. **(**A**) GIP mRNA expression was higher in the five isolated K-cell clones than in STC-1 cells as a control. (**B**) GLP-1 mRNA expressions was higher in the five isolated L-cell clones than in STC-1 cells as control. BioRad CFX Manager Software was used to analyze expression data. Gene expression was normalized for mouse β-actin and β-2 microglobulin (β2m) mRNA expression. Error bars indicate standard deviation.

To confirm that the isolated cells were indeed K and L-cells, we performed Q-PCR to detect GIP and GLP-1 mRNAs. In this analysis, we compared mRNA expression levels in five isolated clones with those of STC-1 cells as a control. It was expected that the isolated cells would show greater mRNA expression of GIP or GLP-1 than STC-1 cells because these incretins are specifically expressed in K and L-cells, respectively, and not in other intestinal cells, and because the STC-1 cell line contains just 7% K-cells and 5% L-cells [25]. As expected, Q-PCR showed that, in identical culture conditions, GIP mRNA expression was greater in all five K-cell clones than in the STC-1 cells (Figure 1a). However, GIP mRNA expression was not equal among the five clones. As shown in Figure 1a, GIP mRNA expression was highest in clone 1 (K-1) and lowest in clone 5 (K-5) in the same experiment. Similar results were observed for the isolated L-cell clones (Figure 1b) [22], where clone 2 (L-2) showed the highest GLP-1 mRNA expression compared with the other clones.

Several techniques have been developed to isolate a specific cell type from a heterogeneous population of cells. Some methods exploit differences in the physical properties of cells, such as the size or density, to differentiate cell types. For example, leukocytes and erythrocytes have different densities and can therefore be separated by density gradient centrifugation. For cell types with a similar size and density, flowcytometry is better able to separate each cell type. In this technique, the cells are separated based on their expression of one or more specific surface proteins that specifically bind to corresponding monoclonal antibodies [26]. Unfortunately, neither of these methods is suitable for gut K and L-cells, as their size, density, and surface proteins are similar to those of other intestinal cells. Therefore, the antibiotic selection method is the most reliable strategy to isolate these cells from the heterogeneous STC-1 cell population. Isolating a pure cell line is vital to establish useful target cells for gene expression studies and compare transcription levels. Pure cell lines are also essential for the study of post-translational processing and protein modifications because pure cells lack interference from surrounding cells.

In the present study, we repeated the Q-PCR assay for isolated K and L-clones twice. The graph in Figure 1 shows that the mean mRNA expression for each clone was not significantly different. All of the isolated clones expressed the specific RNA (i.e., GIP in K-cells and GLP-1 in L-cells) at a greater level than did STC-1 cells. Based on these results, we selected and propagated the K-1 and L-2 clones because their mRNA expression levels of GIP and GLP-1, respectively, were greater than those of the other clones.

### Isolation of insulin-producing K and L-cells

Insulin-producing K-cells were established by transfecting the isolated K-1 clone with the GIP/Ins/pBud plasmid. This plasmid includes an insulin gene under the control of a GIP promoter. This plasmid contained the zeocin resistant gene to select stably transfected cells following treatment with zeocin. The same approaches were used to develop insulin-producing L-cells [22]. The zeocin-resistant clones were isolated and used in insulin expression studies. Insulin expression was determined by Q-PCR assay to measure mRNA expression and by ELISA to measure protein expression. The results of these analyses showed that both cell lines are capable of producing active insulin. By comparing insulin gene expression under the control of different promoters in each cell type also allowed us to determine which of these enteroendocrine cell types is more efficient and suitable for use as host cells for diabetes gene therapy.

### Insulin secretion from K and L-cells in response to glucose and meat hydrolysate

**Figure 2:**
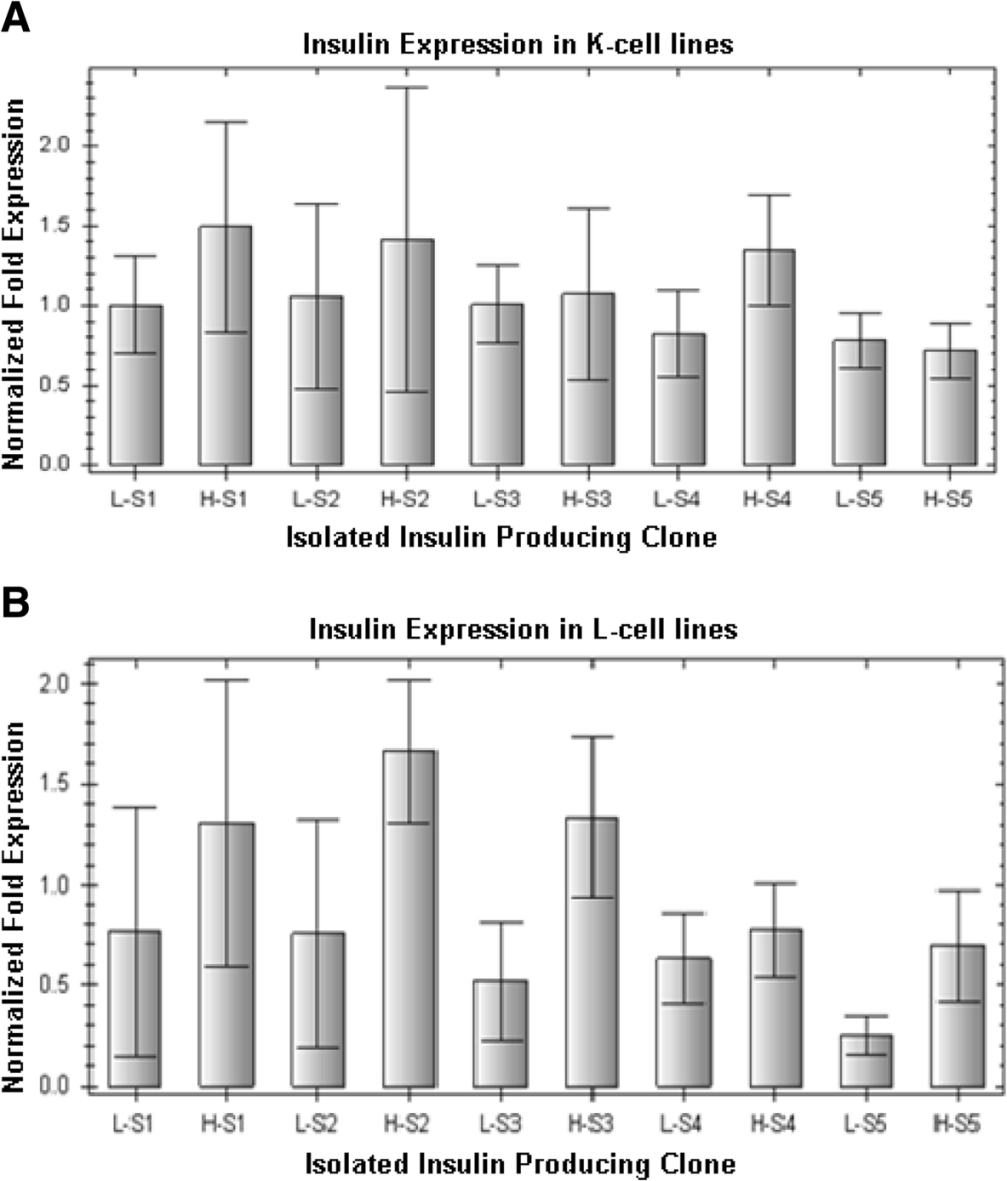
**Q-PCR analysis of insulin mRNA expression.** Insulin expression in the five isolated K-cell clones (**A**) and the five isolated L-cell clones (**B**) was measured following exposure to low (L, 5 mM) or high (H, 25 mM) glucose. L-S1–L-S5: clones 1–5 exposed to low glucose. H-S1–H-S5: clones 1–5 exposed to high glucose. Gene expression was normalized for mouse β-actin and β-2 microglobulin (β2m) mRNA expression. Error bars indicate standard deviation.

The ability of the GIP and GLP-1 promoters to regulate insulin gene transcription was determined by Q-PCR. The five insulin-producing K and L-cell clones were exposed to low or high glucose concentrations. Total RNA were extracted from the cells following treatment for Q-PCR analysis. Consistent with the results of previous studies that compared GIP or GLP-1 promoter activities [17,18,22], we found no differences in human insulin mRNA expression between the high and low glucose conditions (Figure 2). These results indicate that insulin gene transcription under the control of the GIP and GLP-1 promoters is independent of changes in glucose concentration in the culture medium, and that an increase in glucose concentration does not significantly influence promoter activity *in vitro*. The reasons behind it are still unclear but it seems that the responsiveness of the K and L-cells to glucose concentration change is probably due to the glucose detector system in these cells, not because of the transcriptional regulation pathways at the RNA level. Although the activities of the GIP and GLP-1 promoters were not responsive to the change in glucose concentrations, we used these promoters to construct plasmids expressing the insulin gene. The specificity of the GIP and GLP-1 promoters allows selective expression of the insulin gene in the K and L-cells, respectively.

**Figure 3:**
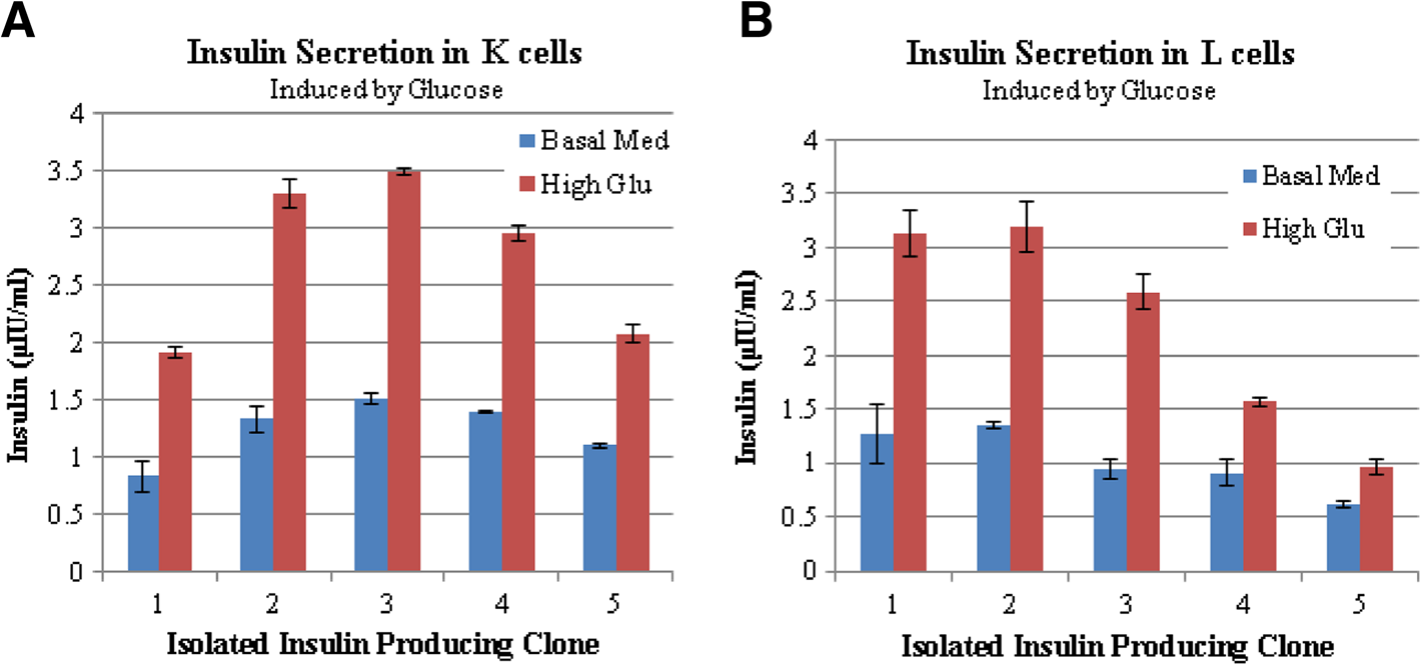
**Effects of glucose stimulation on insulin secretion from K and L-cells.** Insulin secretion was measured in five K-cell clones (**A**) and five L-cell clones (**B**) in response to low (basal, 5 mM) or high (final, 25 mM) glucose. Cell supernatants were collected and insulin was measured by an ELISA. Insulin secretion increased in each of the isolated clones in response to high glucose. Error bars indicate standard deviation.

**Figure 4:**
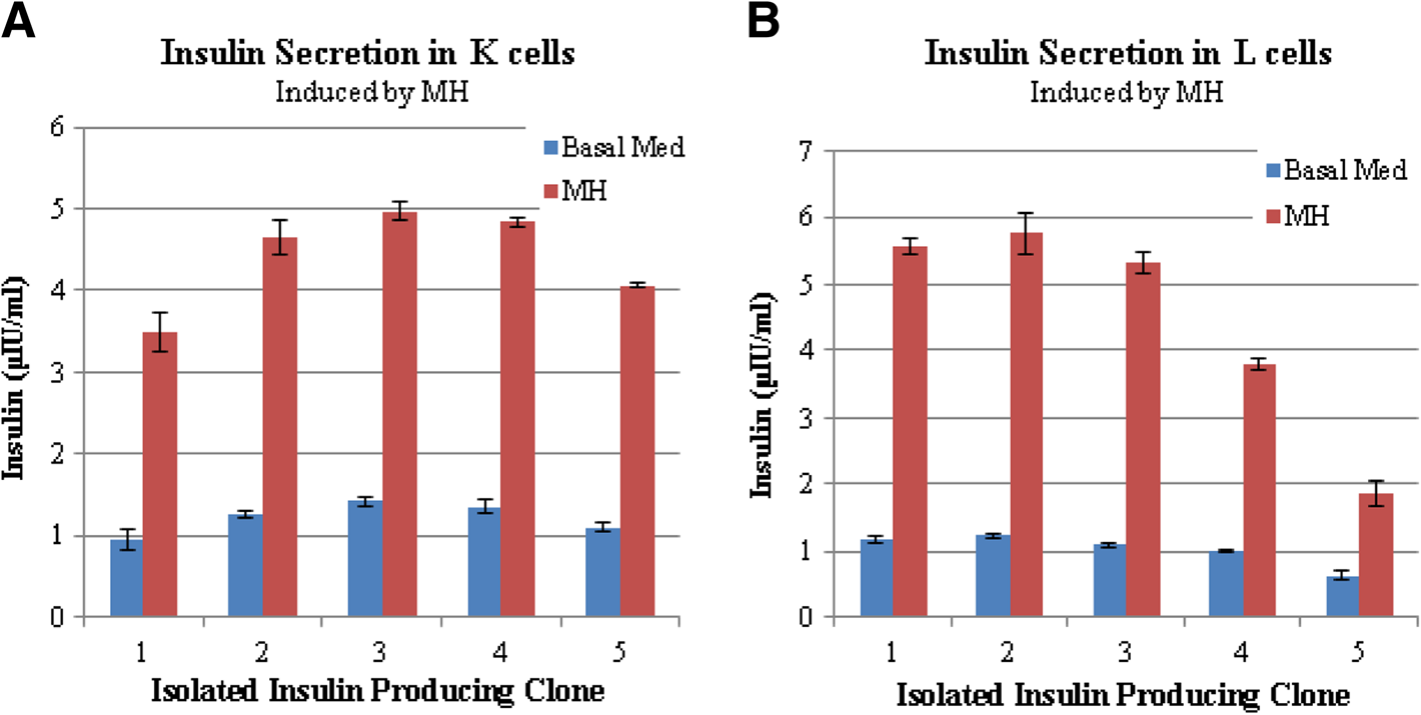
**Effects of meat hydrolysate stimulation on insulin secretion from K and L-cells.** Insulin secretion was measured in five isolated K-cell clones (**A**) and five L-cell clones (**B**) in response to basal medium or meat hydrolysate (MH) (2%, w/v). Cell supernatants were collected and insulin was measured by an ELISA. Insulin secretion increased in each of the isolated clones in response to meat hydrolysate. Error bars indicate standard deviation.

The insulin protein secretion in response to the glucose induction was studied in insulin producing K and L-cells by ELISA test. The results of the ELISAs showed that increasing the glucose concentration from 5 mM to 25 mM increased insulin secretion in all insulin-producing K and L-cells (Figure 3). As shown in Figure 3a, insulin secretion in clones 2 (Ins.K-2) and 3 (Ins.K-3) were increased by about 2.4 fold, reaching 3.29 μIU/ml and 3.49 μIU/ml, respectively. Meanwhile, insulin secretion in L-cells increased by 2.7 fold after glucose induction (Figure 3b) [22]. We also examined insulin secretion in response to 2% meat hydroslyate (Figure 4), which contains a mixture of free amino acids and peptides. Exposure to meat hydrolysate increased insulin secretion by 4.6 fold in L-cell clone 2 (Ins.L-2) and by 3.7 fold in K-cell clone 3 (Ins.K-3). Therefore, exposure to meat hydrolysate markedly increased insulin secretion in both cell types, consistent with the results of earlier studies [16,17,20].

Reviewing Q-PCR and ELISA data, it seems that insulin expression in K and L-cells is regulated by glucose regulated at the protein level but not at the mRNA level. The findings may explain the inability of the GIP and GLP-1 promoters to sense changes in glucose levels. Instead, the intestinal K and L-cells may sense changes in glucose level through existing glucose-sensitive pathways, such as glucokinase and glucose transporter II, which ultimately promote insulin secretion into the medium but not gene expression. Nevertheless, our results confirmed that the engineered gut K and L-cells could secrete insulin in response to stimulation with glucose or meat hydrolysate.

### Comparison of the insulin-producing ability of K and L-cells

Insulin secretion from K and L-cells was markedly increased by exposure to high glucose (25 mM) or meat hydrolysate compared with the basal state (5 mM glucose). Comparison of basal and stimulated insulin secretion between the two cells was statistically significant for both glucose and meat hydrolysate (p < 0.001). As shown in Table 1, exposure to high glucose or meat hydrolysate significantly increased insulin secretion from K-cells, as compared with the basal levels. High glucose and meat hydrolysate had similar effects on insulin secretion in L-cells. These results indicate that the K and L-cells have a functional nutrient sensing system that can respond to different nutrients.

**Table 1:** Effects of high glucose and meat hydrolysate on mean insulin secretion in K and L-cells

**Cell**	** Stimulant**	**n**	**Mean(S.D) μIU/ml**	**Mean diff. (CI95%)**	**Mean% incr. (S.D)**	**t-stat (dF)**	**p-value** *
K-cells	Basal	20	1.22 (0.44)	−1.50	144 (96)	−9.96 (19)	<0.001
	High glucose	20	2.72 (0.75)	(−1.81,-1.18)			
	Basal	20	1.20 (0.45)	−3.18	312 (172)	−20.88 (19)	<0.001
	High MH	20	4.39 (1.21)	(−3.50, -2.86)			
L-cells	Basal	20	1.00 (0.58)	−3.43	776 (1250)	−21.31 (19)	<0.001
	High Glucose	20	4.43 (0.60	(−3.77,-3.09)			
	Basal	20	1.02	−3.41	410 (271)	−10.40 (19)	<0.001
	High MH	20	4.42	(−4.09,-2.72)			

*paired t-test (p-value <0.05 is significant).

We next compared the effects of glucose and meat hydrolysate on the percentage increase in insulin secretion relative to basal levels in both cell types. As shown in Table 2, the percentage increase in insulin secretion was significantly greater following exposure to meat hydrolysate than high glucose in both K-cells (p = 0.001) and L-cells (p = 0.024). We suspect that meat hydrolysate has a greater stimulatory effect than glucose on insulin secretion because it consists of multiple amino acids and fatty acids, which may have greater stimulatory factors compared with glucose. Notably, it has been reported that fat, carbohydrate, protein, and mixed amino acids can stimulate GLP-1 secretion from L-cells and GIP secretion from K-cells [27,28].

**Table 2:** Comparison of the effects of glucose and meat hydrolysate on the percentage increase in insulin secretion in K and L-cells

**Cell type**	**Stimulant**	**n**	**Mean% increase (S.D)**	**Mean diff. (CI95%)**	**p-value** *
K-cells	Glucose	20	144 (95)	−168 (−258,-78)	0.001
	MH	20	312 (172)		
L-cells	Glucose	20	222 (232)	−188 (−349,-26)	0.024
	MH	20	410 (271)		

*independent t-test (p-value <0.05 is significant).

We then sought to determine which cell line shows the best responses to each stimulus and hence provide the more suitable host for gene therapy. Accordingly, we compared the percentage increase in insulin secretion between K-cells and L-cells. Interestingly, these comparisons revealed no significant differences in the responses of K and L-cells to high glucose (p = 0.176) or meat hydrolysate (p = 0.182) (Table 3).

**Table 3:** Comparison of the percentage increase in insulin secretion between K and L-cells treated with high glucose or meat hydrolysate

**Stimulant**	**Cell type**	**n**	**Mean% increase (S.D)**	**Mean diff. (CI95%)**	**p-value** *
High Glucose	K-cells	20	144 (95)	−78 (−194,32)	0.176
	L-cells	20	222 (232)		
MH	K-cells	20	313 (172)	−98 (−244,48)	0.182
	L-cells	20	410 (271)		

*independent t-test (p-value <0.05 is significant).

Finally, we conducted multivariate analysis with the percentage increase in insulin secretion as the dependent variable. The final model included both cell type and stimulant type, which together explained about 18% (R^2^ = 0.18) of the variation in the percentage increase in insulin secretion (Table 4). After controlling for stimulus, the percentage increase in insulin secretion was slightly higher in L-cells than in K-cells (316% vs. 228%), although this was not statistically significant (p = 0.056). Since the p-value was very close to the pre-specified α (0.05), it is possible the sample size was too small to detect this difference. It is reasonable to expect that the responses of K and L-cells to specific nutrient and its levels are not equal. It is possible that in physiological conditions, other elements or factors that are otherwise absent in cell culture conditions might influence insulin secretion.

**Table 4:** Multivariate analysis of the percentage increase in insulin secretion

**Cell type**	**n**	^**a**^Adjusted mean Of% insulin increase (CI95%) *	^**b**^**Adjusted mean diff. Of% insulin increase(CI95%)** †	**F-stat(dF)**	**p-value**
K-cells	40	228 (165,292)	−87 (−178,2)	3.8 (1,77)	0.056
L-cells	40	316 (253,380)			

The final model included cell type and stimulant type as independent factors (p < 0.05; R^2^ = 0.18).

*Adjusted mean (two-way ANOVA controlling for type of stimulant).

†With Bonferroni adjustment of the 95% CI.

## Conclusion

In conclusion, nutrients are an important signal that can stimulate both K and L-cells through direct contact with these cells. K and L-cells show different responses to different nutrients, although their responses were greater to meat hydrolysate than to glucose. Nevertheless, the relative insulin secretion from K and L-cells in response to both stimuli was not markedly different. These results provide compelling evidence supporting the use of both cell types as suitable candidates for diabetes gene therapy and warrant further studies to examine their clinical applications. To avoid erroneous conclusions, the same results should be reproduced *in vivo*, as many therapies that are effective *in vitro* are ineffective *in vivo* for a variety of reasons. The main limitations of *in vitro* studies include the proposed delivery system and toxicity against the host, which was not evident *in vitro*. Future studies should provide more insight into the potential use of both cells for *in vivo* gene therapy or as ex vivo insulin secreting cells for use as an ectopic transplant. Studies are also necessary to confirm that our exciting results can be repeated in animal models. It is also important to identify factors (e.g., neural elements and hormones) that may interfere with insulin gene expression in physiological conditions when using these cell lines.

## Abbreviations

DMEM: Dulbecco’s modified Eagle’s medium; ELISA: Enzyme-linked immunosorbent assay; FBS: Fetal bovine serum; GLP-1: Glucagon-like peptide-1; GIP: Glucose-dependent insulinotropic polypeptide; Q-PCR: Quantitative-PCR; MTT: 3-(4,5-dimethylthiazol-2-Yl)-2,5-diphenyltetrazolium bromide.

## Competing interests

The authors declare that they have no competing interests.

## Authors’ contributions

ZA lead the project, helped to design the project, and helped to write and edit the manuscript. MR helped to design the project, carried out laboratory studies, and drafted the manuscript. AZFA conducted statistical analysis. ARO helped to design the project. All authors read and approved the final manuscript.
